# A critical view of safety in left colectomy surgery: A case of renal artery injury

**DOI:** 10.1016/j.ijscr.2021.106035

**Published:** 2021-05-27

**Authors:** Carmelo Mazzeo, Francesca Viscosi, Giorgio Badessi, Eugenio Cucinotta

**Affiliations:** Department of General and Emergency Surgery, Policlinico G. Martino, University of Messina, Italy

**Keywords:** IMA, inferior mesenteric artery, IMV, inferior mesenteric vein, POD, post operative day, Critical view of safety, Renal artery, Iatrogenic injury, Case report

## Abstract

**Introduction:**

The standardization of the laparoscopic approach in left hemicolectomy was facilitated by the vascular anatomy of the left colon, which has few anatomical variants. The current technique for left hemicolectomy consists in approaching the inferior mesenteric artery (IMA), after identification of the inferior mesenteric vein (IMV), from above (craniocaudal) or from below (caudocranial). The type of approach is decided on the basis of the vascular window between the IMV and IMA. However, vascular abnormalities of adjacent organs can call into question the steps of the standardized technique.

**Case presentation:**

We describe a case of iatrogenic left renal artery injury caused during left laparoscopic hemicolectomy due to an abnormality of the renal vessels. The artery originated from the aorta more caudally than usual with respect to the normal population.

**Discussion:**

What happened made us question the security of the standardized approach in practice, especially in patients with vascular anomalies. The use of advanced imaging programs, such as 3D reconstruction, can help to prevent iatrogenic damages, but not all hospitals have such technology, especially in rural areas.

**Conclusion:**

We propose, to further decrease the risk of iatrogenic injuries, a “critical view of safety” for left colic surgery, in which, before any potential arterial resection, a careful craniocaudal and caudocranial dissection of the Toldt-Gerota plane could be useful in identifying the IMA at the center of this plane. Moreover, a preoperative imaging study is of paramount importance in all surgical procedures.

## Highlights

• The anatomical variations of the left renal vessels may be the cause of iatrogenic vascular lesions in left hemicolectomy.
• Advanced imaging programs and preoperative study can help prevent iatrogenic injuries
• A careful cranio-caudal and caudo-cranial dissection of the Toldt-Gerota plane could identify the inferior mesenteric artery.

## 1 Introduction

Laparoscopic approach to cancers of the left colon has proved valid for several years from the oncological point of view; having the advantages of a faster recovery, better aesthetic result and fewer wall complications compared to the open approach [1]. Over the years, the key steps of the procedure have changed. At the dawn of the laparoscopic technique, surgeons tried to reproduce the laparotomic approach with laparoscopy, following the same steps of the open technique, which involves a lateral-to-medial access to the organ. Today laparoscopic technique prefers the medial-to-lateral approach, which involves an initial identification and section of the vascular structures of the colon. Following this approach, the inferior mesenteric vein (IMV) is the first vascular landmark to be found [2]. The following maneuver consists in the dissection of the inferior mesenteric artery (IMA), which should be the only artery originating from the anterior aspect of the sub-renal aorta. There are two possible ways to identify the IMA. The first is the craniocaudal detachment of the Toldt-Gerota plane below the IMV. The second approach consists of the detachment of the same plane in a caudocranial sense starting from the aortic bifurcation. Some authors choose between the first and the second approach based on the extent of the vascular window between the IMV and IMA. When the window is wide enough, they start from above; when it is less than 5 cm the dissection starts from the bottom instead [3]. However, this technique does not take into account any potential anatomical variation that may be responsible for errors in the identification of the IMA. In fact, while the left colic vessels are constant in their origin and course, this is not absolute for the renal vessels. These variations are present, with a frequency of 1/6 patients [4] (Table 1), and can be considered an important risk factor for patients undergoing abdominal surgery [4,5]. We present a case report of a patient with a vascular variation who underwent surgery for an occluding carcinoma. This case report has been written following the SCARE 2020 criteria [6].

**Table 1:** Distribution of renal vascular anomalies (4).

Renal vessels	Percent
Supernumerary renal vascular structure	4.8
Draining of renal vein into inferior vena cava more distally	3.9
Retro-aortic left renal vein	3.1
Originating of renal artery from aorta more distally	1.3
Precaval right renal artery	1.3

## 2 Presentation of case

**Fig. 1:**
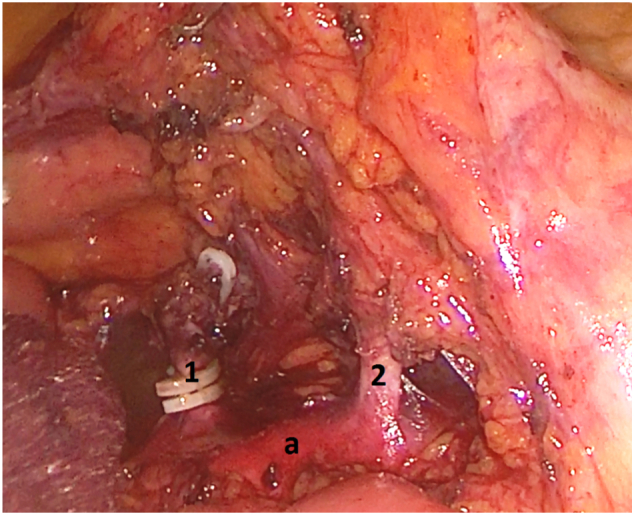
Intraoperative image of the anomalous origin of Renal Artery (1) and the normal position of IMA (2) that arise from aorta (a).

**Fig. 2:**
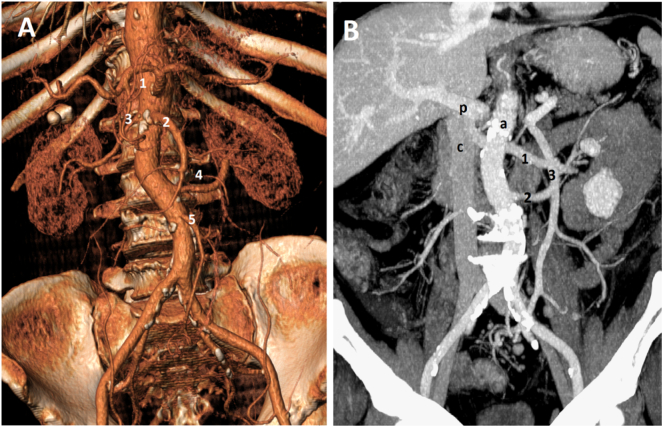
In the three-dimensional CT angiography (A) is highlighted the distal origin of the left renal artery (4) and the normal origin of the other vascular structures: (1) celiac trunk, (2) mesenteric superior artery, (3) right renal artery, (5) inferior mesenteric artery. The CT-scan (B) underlines the anomalous distance between the left renal vein (1) and left renal artery (2). P) portal vein; a) aorta; c) vena cava; 3) mesenteric inferior vein.

A Caucasian 72-year-old man arrived at our emergency room complaining of abdominal pain and evacuation of stools mixed with blood for a couple of days. A CT scan was performed which reported an occluding neoplasia of the sigma, with no distant lesions to other parenchymal organs. At admission the physical examination revealed tenderness in all the abdominal districts, especially in the left iliac fossa; no signs of peritonism were detected. The patient was a smoker, without a history of alcohol abuse, with a history of arterial hypertension treated with one anti-hypertensive drug. Family history was negative for any kind of cancer. Preoperative exams were normal, except for a decrease in hemoglobin (10 g/dl). Biopsy of the mass through rectoscopy was performed, which was compatible for adenocarcinoma of the sigma. We decided to proceed with laparoscopic resection of the tumor. During surgery, according to the standardized technique after IMV identification, we searched for the IMA along a craniocaudal dissection of the Toldt-Gerota plane. Along this dissection, we found a vascular structure that was identified as the IMA and dissected between clips. Later we continued the dissection of the Toldt-Gerota plane in a medial-to-lateral direction from the artery stump, revealing that the course of the dissected structure passed behind the left gonadal vessels. Thus, we realized that the sectioned artery could not be the IMA. To confirm this hypothesis, we went back to seek for the IMA, starting from the aortic bifurcation in a caudocranial direction, and noticed another vascular structure originating from the aorta (Fig. 1). The surgery ended with a conversion to a laparotomic technique, renal artery re-implantation (aortic renal anastomosis latero-terminal) and left hemicolectomy. An intraoperative doppler showed the presence of an adequate renal artery flow. The operation took 210 min, the amount of blood loss was 50 ml. On histological examination of the sigmoid colon, adenocarcinoma was confirmed without lymph node metastasis on the 42 lymph nodes analysed (pT3N0M0, stage IIA). The patient developed a worsening of the renal function, which returned to normal on post-operative day 6. A re-evaluation of the preoperative CT scan confirmed the anatomical variation, revealing a left renal artery originating between L3–L4 (See Fig. 2). The patient was dismissed on the 7th POD, when renal function returned to normal. The oncologist decided not to give chemotherapy on this patient due to the absence of local and distant metastases. A follow-up visit 1 week from discharge showed no particular abnormalities at physical examination and normal lab values. During 12 and 24-month follow-up visits no kind of long-term complication or distant metastases were reported. The patient, despite the iatrogenic injury, was still pleased by the procedure and by the overall treatment. Post-intervention considerations were that a better preoperative study could have given us the chance to better prepare for this kind of procedure. However, since an advanced study of the images is not always available, especially in emergency settings, an alternative approach to this kind of technique should be considered.

## 3 Discussion

The clinical case highlights how the standardized technique with a single direction approach to the IMA, cranial or caudal, does not prevent from a iatrogenic injury of the renal vessels in case of anatomical variation. Generally, the left renal vessels, both artery and vein, are located on a higher plane compared to that of our patient. Normally the left renal artery arises from the aorta just inferior to the origin of the superior mesenteric artery and passes posterior to the left renal vein. In our case the caudal origin of the renal artery, unusually distant from the homolateral renal vein (5.06 cm below the renal vein) was the cause of misidentification of the vessels, which led to the surgical error. This vascular variation constituted, therefore, an incorrect anatomical landmark in the identification of the right surgical plane. A preoperative CT scan showed no anatomical variation of the left colon vasculature and a vascular window of around 5 cm. In interpreting the images, we did not pay attention to the direction and origin of renal vessels, overlooking a distal origin of the left renal artery, in respect to the renal vein. An approach to the IMA starting from the bottom would probably have allowed us to avoid this complication. However, if the renal artery originated even lower, the injury could have occurred anyway. In literature there are few descriptions of iatrogenic vascular injuries during left hemicolectomy. Kazushige Kawai et al. reported a case of left renal vein originating from the left iliac vein during left hemicolectomy. In that case no vascular injuries were reported [7]. Kentaro Saito et al. described a case, similar to our clinical case, with a caudal left renal artery developing from the abdominal aorta on the caudal side of the root of the IMA [8]. The conclusion of the studies cited above is that a vascular preoperative assessment with the use of a three-dimensional CT angiography is necessary in order to prevent any iatrogenic damage. However, this imaging technology is not available in all surgical units. What we propose, to minimize the risk of vascular injuries during left hemicolectomy, is to implement a “critical view of safety” in the colic setting. Strasberg et al. showed that to reduce the risk of biliary injuries during cholecystectomy, the cystic artery and duct dissection should be made only after excluding the presence of other structures originating from the gallbladder [9]. In the same way we propose, before clipping and sectioning the IMA, to always prepare both cranial-caudally and caudo-cranially the Toldt-Gerota plane, in order to identify only one vascular structure arising from the abdominal aorta heading to the left colon. Moreover, it is advisable to verify the course of the isolated structure in relation to the other structures present in the Gerota plane, such as the ureter, renal and gonadal vessels.

## 4 Conclusions

The anatomical variations of the left renal vessels may be the cause of iatrogenic vascular injuries, during left hemicolectomy. Thus, to minimize this risk, we propose a critical view of safety for laparoscopic left hemicolectomy: a careful dissection of the Toldt-Gerota plane, conducted both along a craniocaudal and caudocranial fashion, should be performed to identify a unique structure originating from the abdominal aorta at the center of this dissection. Hence, identification and isolation of the inferior mesenteric artery, as the sole tubular structure present in the middle of our Toldt-Gerota dissection, needs to be completed before any vascular dissection. If more structures are present, any vascular section should be avoided before the correct identification of the IMA. Moreover, before starting the procedure, a thorough study of the CT scan images, especially of the most important vessels, should be conducted in order to anticipate any kind of anatomical variation.

## Sources of funding

None declared.

## Ethical approval

N/A.

## Consent

Written informed consent was obtained from the patient for publication of this case report and accompanying images. A copy of the written consent is available for review by the Editor-in-Chief of this journal on request.

## Author contribution

All authors contributed equally.

## Research registration (for case reports detailing a new surgical technique or new equipment/technology)

N/A.

## Guarantor

Giorgio Badessi.

## Provenance and peer review

Not commissioned, externally peer-reviewed.

## Declaration of competing interest

None declared.
